# Immunotherapy Assessment: A New Paradigm for Radiologists

**DOI:** 10.3390/diagnostics13020302

**Published:** 2023-01-13

**Authors:** Vincenza Granata, Roberta Fusco, Sergio Venanzio Setola, Igino Simonetti, Carmine Picone, Ester Simeone, Lucia Festino, Vito Vanella, Maria Grazia Vitale, Agnese Montanino, Alessandro Morabito, Francesco Izzo, Paolo Antonio Ascierto, Antonella Petrillo

**Affiliations:** 1 Division of Radiology, Istituto Nazionale Tumori IRCCS Fondazione Pascale—IRCCS di Napoli, 80131 Naples, Italy; 2 Medical Oncology Division, Igea SpA, 80013 Naples, Italy; 3 Italian Society of Medical and Interventional Radiology (SIRM), SIRM Foundation, 20122 Milan, Italy; 4 Melanoma, Cancer Immunotherapy and Development Therapeutics Unit, Istituto Nazionale Tumori IRCCS Fondazione G. Pascale, 80131 Naples, Italy; 5 Thoracic Medical Oncology, Istituto Nazionale Tumori IRCCS Fondazione Pascale—IRCCS di Napoli, 80131 Naples, Italy; 6 Division of Epatobiliary Surgical Oncology, Istituto Nazionale Tumori IRCCS Fondazione Pascale—IRCCS di Napoli, 80131 Naples, Italy

**Keywords:** immunotherapy, radiological response assessment, Recist 1.1, i-Recist, immuno-related adverse events

## Abstract

Immunotherapy denotes an exemplar change in an oncological setting. Despite the effective application of these treatments across a broad range of tumors, only a minority of patients have beneficial effects. The efficacy of immunotherapy is affected by several factors, including human immunity, which is strongly correlated to genetic features, such as intra-tumor heterogeneity. Classic imaging assessment, based on computed tomography (CT) or magnetic resonance imaging (MRI), which is useful for conventional treatments, has a limited role in immunotherapy. The reason is due to different patterns of response and/or progression during this kind of treatment which differs from those seen during other treatments, such as the possibility to assess the wide spectrum of immunotherapy-correlated toxic effects (ir-AEs) as soon as possible. In addition, considering the unusual response patterns, the limits of conventional response criteria and the necessity of using related immune-response criteria are clear. Radiomics analysis is a recent field of great interest in a radiological setting and recently it has grown the idea that we could identify patients who will be fit for this treatment or who will develop ir-AEs.

## 1. Background

Immunotherapy denotes an exemplar change in an oncological setting [1,2,3,4,5,6]. In fact, different to other therapies as conventional chemotherapy [7,8,9,10,11,12,13], such as radiation [14,15,16,17,18,19,20] or targeted therapies [21,22,23,24,25,26,27] which target the cancer, these treatments work by stimulating the patient’s immune system to obtain an immune reaction against the tumor [2,3].

Immunotherapy can be categorized as passive or active, according to the action mechanism [28,29,30,31,32,33,34]. In passive treatment [28,29,30], immunoglobulins can be administered and attached to tumor-related antigens. Otherwise, in the active treatment, there is a stimulation of the immune system to recognize antigens of the tumor and act against them [30,31,32,33,34]. Although several approaches are presently utilized in clinical and pre-clinical settings, the main means are centered on the so-called checkpoint inhibitors (ICI), that include programmed cell death–1 (PD-1) protein, PD-1′s main ligand (PD-L1), and cytotoxic T-lymphocyte–associated protein 4 (CTLA-4) [35,36,37,38,39,40,41,42,43].

In 2011, the U.S. Food and Drug Administration (FDA) approved the first agent, ipilimumab, a CTLA-4 inhibitor, for metastatic and unresectable melanoma [44]. Subsequently, different FDA approved agents, have been introduced in both routine and experimental studies to treat different categories of solid and hematologic tumors as after failure of conventional therapies or as first-line therapies [39,45,46,47,48,49,50,51,52,53,54,55,56,57,58,59,60,61,62,63,64,65,66,67,68,69]. Moreover, these treatments may be combined with conventional treatments since they can increase the cytotoxic effect of chemotherapy [70,71,72].

Although the effective utility of these treatments is within a wide range of tumors, only a sub-group of patients show real benefits [73]. The efficacy of immunotherapy is affected by several factors, including human immunity, which is strongly correlated to genetic features [74,75,76,77], such as intra-tumor heterogeneity [78,79,80,81,82,83,84,85,86]. In fact, cancer is complex, flexible, and heterogeneous, representing the result of an innumerable number of genetic mutations that affect the regular cell functionality. However, these mutations allow the cancer cell to appear as foreign to the immune system, offering an opportunity for this treatment [78,79,80,81,82,83,84,85,86]. Clinical studies have demonstrated that even when several patients are well qualified as immunotherapy responders (e.g., high tumor PD-L1 expression), a large percentage of them (>50%) do not respond to this [87]. In addition, during treatment, some patients can have a clinical and/or radiological disease progression [88]; these events are known as “primary immune escape” and “secondary immune escape”, respectively [89]. The mechanisms implicated in these two phenomena (also known as resistance) can overlap. Multi-omics analyses obtained from immunotherapy-treated patient tissues showed as several features are correlated to immune resistance [90,91,92].

In addition, while immunotherapy has increased patient outcomes, it can cause several immune-related adverse events (ir-AEs), which can be transitory or chronic, life-threatening or mild, and could affect several organ systems, sometimes numerous organs at the same time [93,94,95,96,97,98,99,100,101,102,103,104,105,106,107,108].

Classic imaging assessment, based on computed tomography (CT) [109,110,111,112,113,114,115,116] or magnetic resonance imaging (MRI) [117,118,119,120,121,122,123,124,125,126,127], which is useful for conventional treatment, has a limited role in immunotherapy. The reason is due to different patterns of response and/or progression during this kind of treatment which differs from those seen during other treatments, such as the possibility to assess the wide spectrum of immunotherapy-related toxic effects as soon as possible [128,129,130,131,132,133,134,135,136,137,138,139]. Thus, the introduction of robust noninvasive imaging biomarkers that can allow the immunotherapy-response prediction and prognosis is crucial.

The aim of this article is to (a) explain the response pattern and response criteria utilized during immunotherapy assessments, (b) explain the wide spectrum of toxic effects due to immunotherapy, and (c) explain the potential role of radiomics features as imaging biomarkers for immunotherapy.

## 2. Treatment Assessment and Pattern Response

The frequently employed radiological response criteria have been introduced considering the chemotherapeutic agents’ cytotoxic results, which caused a decrease in target size when the treatment has been effective [132,133,140]. The World Health Organization (WHO) criteria assessed the tumor burden considering the sum of the products of orthogonal largest diameters of the target lesions [141], while the response evaluation criteria in solid tumors (RECIST) criteria [142] assessed the tumor burden considering a one-dimensional (the largest diameter of target lesions). These criteria are not able to evaluate functional or metabolic status, such as necrosis, that causes morphological changes on CT (density decrease) or MRI (inhomogeneous signal on conventional sequences and different pattern on functional ones) [130,143,144,145,146]. The CHOI criteria [143] were introduced to assess target therapy in gastrointestinal stromal tumors, such as the PET response criteria in solid tumors (PERCIST) to evaluate FDG uptake reduction [147,148].

**Figure 1:**
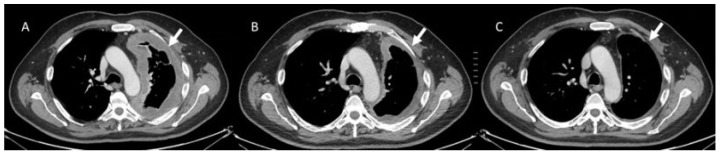
Complete response pattern in a mesothelioma patient treated with immunotherapy. CT scan assessment in pre-treatment phase (**A**) of lesion (arrow), at 3 months (**B**), and after 6 months (**C**).

**Figure 2:**
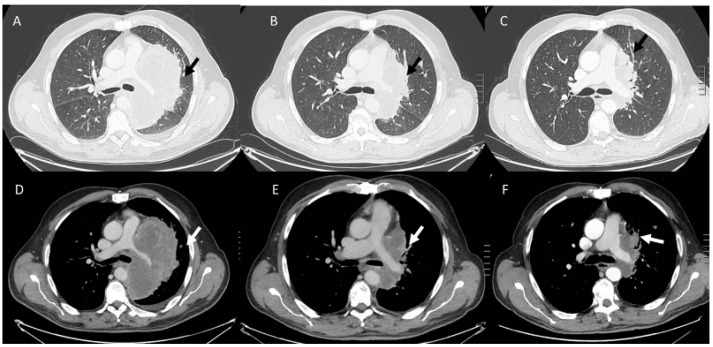
Partial response pattern in non small lung cancer (NSLC) treated with immunotherapy (arrows). CT scan assessment in pre-treatment phase (**A**,**D**), at 3 months (**B**,**E**), and after 6 months (**C**,**F**).

**Figure 3:**
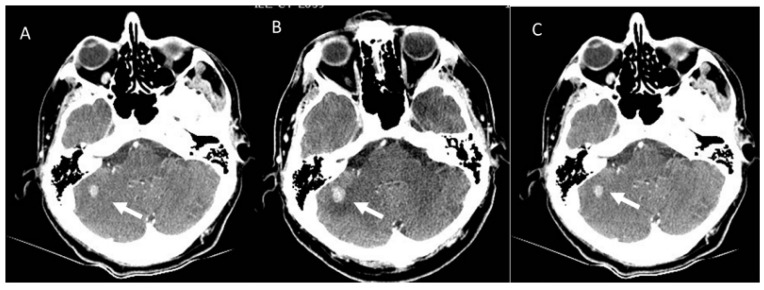
Pseudoprogression of brain metastasis (arrows) in an NSLC patient treated with immunotherapy. CT evaluation (**A**) in pre-treatment phase, after 1 month (**B**), and after 12 weeks of the first CT evaluation (**C**).

**Figure 4:**
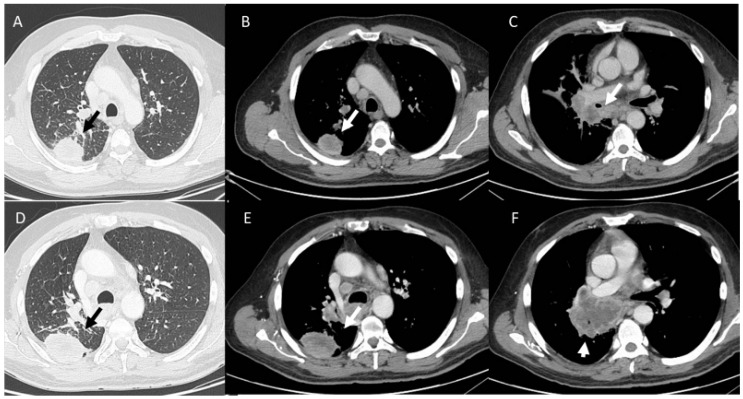
Iperprogression in an NSLC (arrows) patient treated with immunotherapy. CT assessment in pre-treatment phase (**A**–**C**) and after 3 months (**D**–**F**).

**Figure 5:**
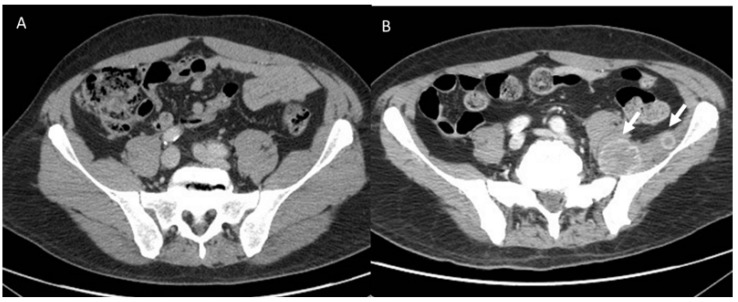
The same patient in Figure 4 (**A**). New lesions (arrows) in ileo-psoas muscle (**B**) due to iperprogression during immunotherapy.

In contrast to conventional treatments, immunotherapy involves a complex process that includes different phases and during each one, the immune system is activated. Such as, a number of immune cells move to the target with increasing in target volume and/or new lesion growth [139,149,150]. This process can cause an unusual response pattern known as pseudoprogression [139]. Although, it is possible to obtain conventional response patterns as a complete response (Figure 1) or a partial response (Figure 2), pseudoprogression (Figure 3), characterized by an increment in volume target, can occur in 4–10% of immunotherapy-treated patients [128]. Divergent to pseudoprogression, in which treatment is preserved, a true severe progression is hyperprogression (Figure 4 and Figure 5) [128]. During an imaging study assessment, it could be complicated differ pseudoprogression to hyperprogression [128], so a multidisciplinary team should assess complete patient status [128].

**Figure 6:**
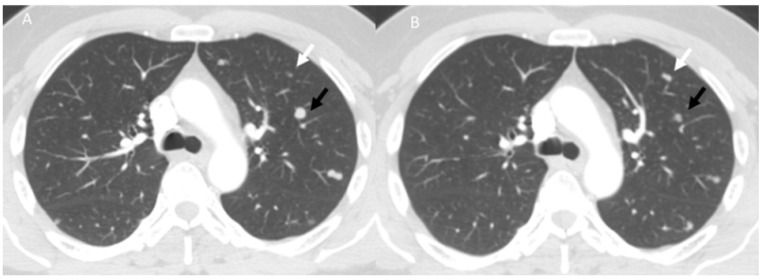
Dissociated responses in a melanoma patient during immunotherapy. CT assessment in pre-treatment phase (**A**) and at 3 months (**B**) follow-up: black arrows show the regression of the lesion while white arrows show the increase in lesion dimensions.

Another atypical response pattern are dissociated responses (Figure 6) [128]. In this pattern, several lesions show dimension increase and others show regression. This pattern is associated with a better survival compared to true progressions [128].

Considering these response patterns, the limits of conventional response criteria are clear. So, to overcome these limits, new criteria have been proposed. The first criteria were the immune-related response criteria (irRC), based on the WHO criteria [136]. In these criteria, two main features were introduced: (*a*) the first radiological progression may be confirmed after 4 weeks, so in this phase, the patient can be treated; (*b*) the development of new lesions is not assessed as a disease progression, but these may be included in the total tumor burden [136]. The main limit of irRC is due to the bi-dimensional measurement of target lesions. In addition, since the greater part of immunotherapy trials in development at that time have been assessed according to RECIST, the obtained results could be difficult to compare according to the new criteria [137]. Subsequently, the immune-related RECIST (irRECIST), established on the unidimensional evaluation [138], have been proposed. According to irRECIST, an increase of 20% in the total tumor burden from nadir with a minimum of 5 mm, such as the progression of non-target lesions or the appearance of a new lesion, has been defined as immune-related progression disease (irPD). Moreover, in this case, the irPD must been verified after 4 weeks, and if there is also a new unequivocal progression (UEP) in this phase, the patient is defined in disease progression [138].

To homogenize results between different trials, the RECIST working group proposed an adaptation to the immunotherapy version of RECIST1.1, the immune RECIST (iRECIST) [139]. The iRECIST identified a standard lexicon including immune complete response (iCR), immune stable disease (iSD), immune PR (iPR), and immune unconfirmed PD (iUPD) or confirmed PD (iCPD). The key element in iRECIST is that iUPD must be confirmed by imaging at least 4 weeks after the first evaluation, but no more than 8 weeks from iUPD, and that a patient may be classified as iUPD more times until there is an iCPD [139]. Regarding new lesions, these must not be involved in the sum of total tumor burden, but the patient can be classified as an iUPD. After an iUPD, the appearance of another new lesion, increase in size of target or non-target lesions, an increase in the sum of measurement of new target lesions >5 mm, or any progression of new non-target lesions can cause an iCPD [139].

Although these immunotherapy-response evaluation criteria have been proposed to allow the comparison of different clinical trial results, at the present, these are not utilized in clinical settings, such as none of these takes into account the atypical response patterns as hyperprogression or dissociated responses.

## 3. Immune-Related Adverse Events Assessment

If on the one hand, immunotherapy has improved oncological patient outcomes, on the other, these treatments have caused a rise in adverse events, called ir-AEs, which can involve several tissues and organs, from head to toe [93,95,96,151,152,153]. The ir-AEs are classified according to the Common Terminology Criteria for Adverse Events (CTCAE v 5.0) [94], which; moreover, permits to assess these events throughout several clinical trials. A systematic review showed that about 74% of patients treated with anti-PD-L-1 inhibitors developed ir-AEs, and among them, 14% had a grade ≥3, while the rate increased in anti-CTLA-4 inhibitors treated patients (about 89% and in this group, about 34% had a grade ≥3). When the authors evaluated patients treated with a combination of ICIs, the rate was 90%, and among them, 55% had a grade ≥3 [95].

The ir-AEs are relatively new conditions, so very little is known about them. The enhancement of the immune system is probably also responsible for an accidental autoimmune response [97,98,99,100,101,102,154,155,156,157,158,159,160]. Recombinant cytokines have a high toxicity profile, with low possibility either in terms of the target population either duration time. Toxic effects can involve several organs, as gastrointestinal tract, liver, lungs, heart, skin, endocrine, and hematologic systems [161,162,163,164]. The ir-AEs can appear early, even after the first dose. During ipilimumab treatment, the majority of ir-AEs appeared around 12 weeks, although the appearance time is due to the target: for skin, around 3 weeks; for the liver, around 3–9 weeks; for the gastrointestinal-tract, around 8 weeks; and for the endocrine system, around 7–20 weeks [165]. The majority of ir-AEs are mild or moderate, and readily reversed by stopping the treatment and starting corticosteroid therapy [128]. Otherwise, endocrinopathy related events are usually irreversible [128]. Life-threatening ir-AEs necessitate hospitalization and, in higher grade, intensive care unit admission [128]. To prevent the risk of milder forms becoming life threatening, radiologists should recognize ir-AEs and should inform the clinical team as soon as possible. Consequently, there is a rising request for radiologists to recognize ir-AEs imaging features to permit proper patient management [97,98,99,100,101,102,154,155].

Regarding ir-endocrinopathies, hypophysitis, occurring in 10–13% of ipilimumab patients, is the most frequent [99,100,101,166,167]. The typical symptoms are headache and fatigue with anterior hypopituitarism and multiple hormonal deficiencies. At MR assessment, the patient shows diffuse enlargement of the pituitary with no optic chiasm compression. It is possible to find pituitary stalk thickening, such as during post-contrast assessment pituitary enhancement [128].

With regard to gastrointestinal ir-AEs, the main symptom is diarrhea in a colitis setting [128]. A study of 162 melanoma ipilimumab treated patients showed colitis in 28 (19%) of them [168]. On CT evaluation, typical colitis features include bowel wall thickening with increased enhancement and mesenteric hyperemia, such as a fluid-filled colon. Considering the extension of the colitis, two patterns have been reported: the diffuse and segmental.

In the diffuse pattern, we find a fluid-filled distended colon with mild diffuse wall thickening and mesenteric vessel engorgement. In the segmental pattern, colitis is associated with pre-existing diverticulosis (SCAD), so that we find a moderate segmental wall thickening and pericolic fat stranding [128].

**Figure 7:**
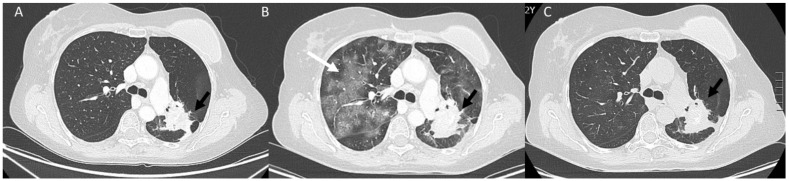
NSLC (black arrows) patient treated with immunotherapy. CT assessment in pre-treatment phase (**A**). During follow-up, in (**B**), appearance of ir-Pneumonitis (white arrow), with disease regression after corticosteroid treatment (**C**).

Pneumonia (Figure 7) is an ir-AE characterized by a focal or diffuse inflammation of the pulmonary parenchyma, and its incidence in immunotherapy-treated patients ranges from 0 to 10% [43]. Compared to pneumonia due to conventional chemotherapy, immunotherapy treated patients showed a greater susceptibility to develop this adverse event, showing an increased risk of high-grade pneumonia, which could cause significant morbidity, possible discontinuation of treatment, and a significant rate of mortality. However, according to previous authors, clinical and radiological diagnosis can improve patient outcomes [102,159,169,170,171,172,173,174,175,176,177,178,179,180,181,182,183,184,185,186,187]. Imaging has a critical role in pneumonitis detection, and CT is the modality that should been chosen since this tool allows the identification of all little changes in the lung and the characterization of different sub-types [179,180,181,182,183,184,185,186,187]. Delaunay et al. [159] identified different patterns on CT studies from 64 patients, (*a*) organized pneumonia (OP) (occurred in about the 23% of patients), (*b*) hypersensitivity pneumonitis (HP) (in 16% of patients), (*c*) non-specific interstitial pneumonia (NSIP) (in 8% of patients), and (*d*) bronchiolitis (in 6% of patients), showing as in the same patients was possible to detect different patterns [159].

Usually, in OPs sub-type, the typical features are bilateral peribronchovascular and subpleural ground-glass and air space opacities, with mid- to lower-lung predominance [159]. CT features in HP pattern include diffuse and predominant ground glass centrilobular nodules in the upper lobe [159]. In NSIP pattern, there are ground glass and lattice opacities prevalently in the lower lobe. Sub-pleural sparing of the posterior and inferior lobes is a specific feature [159]. The bronchiolitis pattern is characterized by centrilobular nodularity, with a tree-in-bud pattern [159]. Acute interstitial pneumonia (AIP)–acute respiratory distress syndrome (ARDS) is not a typical pattern of ir-pneumonitis; however, it is possible to find this sub-type in extensive pulmonary involvement [43].

Granulomatosis and sarcoid-like lymphadenopathies involve mediastinal and hilar lymph nodes [128]. On CT, mediastinal and hilar lymphadenopathies have an appearance and distribution similar to sarcoidosis nodes [128], so it is critical to recognize this condition to avoid defining a PD.

Liver involvement is a rare condition (nearby 1–2% of treated patients) [128]; usually only elevated liver function test results are detected, while in severe cases of ir-hepatitis on imaging tools, it is possible to assess periportal edema, hepatomegaly, and periportal lymphadenopathies [128].

## 4. Radiomics and Immunotherapy

Recently, the idea that imaging studies contain a great amount of data as grey level patterns that usually are imperceptible to the human eyes has become more and more interesting [188,189,190,191,192,193,194,195,196,197,198]. These texture features, when correlated with clinical-pathological data and outcomes [199,200,201,202,203,204,205,206,207,208,209,210,211], theoretically allow for diagnostic and prognostic assessments and it could produce evidence-based clinical-decision support systems [212,213,214,215,216,217,218,219,220]. The assessment of textural characteristics, obtained using radiological images which depend on mathematical analysis as histogram analysis, is called radiomics [221,222,223,224,225,226,227,228,229,230,231,232,233]. The main objective is to combine several multimodal quantitative data with mathematical methods to provide clear and robust parameters allowing an outcome prediction. Radiomics offers outstanding benefits over qualitative imaging assessment since this is clearly limited by the subjective evaluation of radiologists. A radiomics information extension can be obtained by adding genomics data (radiogenomics); in fact, genomic markers, such as microRNA expression, have been shown to be associated with treatment response, metastatic spread, and prognosis that could offer personalized and precision medicine. This approach is captivating since it could be used to obtain molecular data from images [229,234,235,236,237,238,239,240,241,242,243,244,245] with no invasive approach to reduce costs, time, and any risk for the patient. For several tumors, radiomics has already offered a precise molecular assessment, allowing the recognition of biomarkers that are associated with the prognostic assessment [223,224,225,226,227,228,229,230,231,232,233,234,235,236,237,238,239,240].

Tumor immune phenotypes can be classified by histological and immunohistochemical analysis as immune-inflamed, immune-excluded, and immune-desert types [246]. Dense tumor-infiltrating lymphocytes (TILs) and a high tumor mutational burden characterize immune-inflamed tumors. These elements correlate strongly to favor immunotherapy response [247]. Otherwise, in immune-excluded and immune-desert types, which have low TILs and highly proliferating tumor cells, the probability of primary immune escape is high [89]. New biomarkers are needed to quantify TILs and PD-L1 expression to predict and monitor tumor immunotherapy response [248].

Several authors have assessed the radiomics features in predicting response for this treatment [39,249,250,251,252,253,254,255,256]. Granata et al. [39], in lung adenocarcinoma patients, showed that the shift in the center of mass of the lesion was significant as in prediction of overall survival (OS) as in prediction of progression free survival (PFS). By using univariate analysis, the authors obtained low diagnostic accuracy, while by using multivariate analysis, a support vector machine model showed the best results for stratifying patients based on OS (area under curve (AUC) of 0.89 and an accuracy of 81.6%). In addition, a decision tree model showed the best results for stratifying patients based on PFS time (AUC of 0.96 and an accuracy of 94.7%) [39]. Sun et al. [249], in a retrospective multicohort study, analyzed CT images and CD8 T cell RNA expression data from 135 treated patients with anti-PD-1 and anti-PD-L1 antibodies to identify and validate a radiomics signature to predict treatment response. They showed that a high baseline radiomics score was correlated with a higher objective response rate and OS [249]. Moreover, Tang et al. validated a non-small-cell lung cancer (NSCLC) radiomics signature associated with PD-L1 expression and density of TILs [250]. They showed that the radiomics signature was correlated to OS [250]. Tunali et al. [252] demonstrated that a pretreatment radiomics model was able to predict rapid disease progression phenotypes, including hyperprogression (AUCs ranging 0.804–0.865).

The results of these studies, although still in an embryonic phase, could support the hypothesis that radiomics analysis may represent an effective biomarker to select immunotherapy-responsive patients.

In addition, with great interest, we look at the possibility of applying radiomics to the identification of patients who will develop ir-AEs. Colen et al. [253], as the first group, assessed the possibility of radiomics to predict ir-pneumonitis. They obtained radiomics features from a CT study of patients who did (*n* = 2) and did not (*n* = 30) develop this event. However, the major limitation of this study was related to the number of patients and unbalanced groups (*n* = 2 vs. *n* = 30). Larger groups of patients and balanced groups are needed for radiomics analysis.

Thomas et al. [254] assessed 39 patients subjected to chemo-radiation therapy and consolidative ICI therapy for locally advanced NSCLC. They evaluated radiomics data, obtained by pre-treatment [99 mTc] MAA SPECT/CT perfusion images and clinical data. In their study population, 16/39 (41%) patients developed pneumonitis and, with regard to clinical characteristics, only the presence of baseline chronic obstructive pulmonary disease (COPD) was correlated to pneumonitis. Perfused lung radiomics texture features were correlated with lung volume, representing surrogates rather than independent predictors of pneumonitis risk. However, the major limit of this study was related to the number of patients and the lack of external validation group.

Several limitations to the clinical application of radiomics remain. The first key challenge is the use of different imaging techniques by different institutions. To ensure that the academic community can obtain high-quality radiological data resources, it is necessary to establish and promote certain imaging acquisition protocols. Second, the current research uses different software and different feature-selection methods, focuses on different feature sets, and applies different statistical and bioinformatic methods for data analysis and interpretation, which limit the reproducibility of radiomics models. Future research workflows need to be standardized. Third, many relevant radiomics studies employ single-center retrospective datasets. A quality-controlled multicenter prospective study plan is ideal. In addition, the evidence level rating reflects the feasibility of incorporating radiomics research into clinical practice. Recently published guidelines and checklists aiming to improve the quality of radiomics studies, including the radiomics quality score, modified Quality Assessment of Diagnostic Accuracy Studies tool, image biomarker standardization initiative guideline, and Transparent Reporting of a multivariable prediction model for Individual Prognosis or Diagnosis checklist, have been applied to radiomics evaluation [257,258].

## 5. Conclusions

Immunotherapy has a crucial role in the treatment of several tumors. Radiological assessment is essential for evaluating tumor response such as ir-AEs in immunotherapy-treated patients. Knowledge of the current response criteria and the response patterns, such as the different patterns of ir-AES, is critical for radiologists to offer valuable data for clinical providers to guide patient therapies. Radiomics analysis is a promising tool for the selection of responsive patients to immunotherapy such as in early ir-AES detections in order to avoid unnecessary treatments for unfit patients.

## Data Availability

Data are reported in the manuscript and images are reported at link https://zenodo.org/record/7521647#.Y72ceXbMK3A (accessed on 25 October 2022).
